# Combining reinforcement learning and virtual reality in mild neurocognitive impairment: a new usability assessment on patients and caregivers

**DOI:** 10.3389/fnagi.2023.1189498

**Published:** 2023-05-24

**Authors:** Fabrizio Stasolla, Mariacarla Di Gioia

**Affiliations:** ^1^University “Giustino Fortunato” of Benevento, Benevento, Italy; ^2^Universitas Mercatorum, Rome, Italy

**Keywords:** mild neurocognitive impairment, dementia, quality of life, new technologies, caregivers

## Introduction

Mild neurocognitive impairment (MNI) refers to a transitory stage between normal cognitive decline due to progressive aging and an early stage of dementia. It usually includes both subjective troubles and objective diseases higher than those commonly observed in normal aging or for the individual's education level [American Psychiatric Association (APA), 2013]. However, MNI does not negatively interfere with daily activities (Alfano et al., 2022; Ferrer-Cairols et al., 2023). Subjective cognitive decline (SCD) is defined as a personal health and clinical condition in which an individual's self-perception is affected by worsening cognitive abilities and functioning compared to their previous levels of performance (Boccardi et al., 2022; Creavin et al., 2022). The literature available emphasizes that people with SCD have a higher risk for suffering dementia than young and older adults without SCD because their subjective cognitive decline partially reflects a fine disease that does not yet meet the MNI criteria or diagnosis (Bellomo et al., 2022; Weinstein et al., 2022; Bradfield, 2023). In addition to the pharmacological approach, cognitive rehabilitation has been increasingly adopted (Chen et al., 2021; Chae and Lee, 2023; Gómez-Soria et al., 2023; Perra et al., 2023).

Cognitive rehabilitation may be delivered either through traditional strategies (i.e., paper and pencil approach) or *via* computerized options through the development of Information and Communication Technologies (Brandt et al., 2022). Beside assistive technology, which is useful to build a bridge between the individual's functioning and the requirements of their daily environment, new technologies rely on the use of wearable technologies, mobile technologies, serious games, and telerehabilitation (Di Lorito et al., 2021; Bacanoiu and Danoiu, 2022; Robinson and Moghaddam, 2022). Furthermore, virtual reality (VR) setups may be used (Moon and Han, 2022; Zuschnegg et al., 2022; Jang et al., 2023). Computer-mediated interventions can make up for some of the disadvantages of traditional approaches. For example, cost, time, and human resources may be easily managed through computerized systems. Highly motivating instructions may be delivered, and contextual feedback may be provided. Task difficulty, response time, and stimuli delivery may vary frequently and can be customized based on the participant's features and performance (Lasaponara et al., 2021; Lissek and Suchan, 2021; Gambella et al., 2022). Further development of computer-based strategies is represented by artificial intelligence (AI) systems. Among AI, reinforcement learning (RL) is defined as an AI expert system devoted to systematically adapting task complexity by learning and adjusting it on the basis of the participant's performance (Andriella et al., 2020; Hashmi and Barukab, 2023). Thus, an artificial expert agent will continuously calibrate the exercise complexity and difficulty based on the participants' responses (Yamaguchi et al., 2010). Specifically, the agent is reinforced by the participant's performance and learns through it. Accordingly, the agent will adapt to the task complexity. Furthermore, it is a customized and individualized technological solution, capable of constantly adapting to the task difficulty based on the participant's performance (Kubis et al., 2021).

Despite interesting, encouraging, and promising perspectives, existing literature on both topics remains sparse. A search in the Scopus database reveals that only 15 records were available by merging MNI and VR as keywords. By merging MNI and RL, 36 records were found. Nevertheless, only one record was detected if “caregivers” was added as a keyword, and no records were found if “quality of life” was included as an additional keyword. Conversely, once caregivers and quality of life were included in NCI and VR, 14 and 10 records were found, respectively. On the contrary, by considering mild cognitive impairments (MCIs) and VR, a larger literature was found (Moreno et al., 2019; Chen et al., 2021; Colombini et al., 2021; Jonson et al., 2021; Bacanoiu and Danoiu, 2022; Kim et al., 2022; Li et al., 2022). Specifically, Bacanoiu and Danoiu (2022) carefully assessed the suitable intervention patterns, surveys delivered through variable online platforms, and tools to observe the stagnation of early aging and patients with dementia. The importance of telerehabilitation and scheduled physical exercises quantified by specific indicators was recommended. Chen et al. (2021) explored the use of VR on different categories of patients through a meta-analysis. Thirty-five randomized controlled trials of VR-based interventions were compared to conventional training. The experimental groups performed better than the control groups in daily participation in all categories of patients. Because of the controversy created by publication bias, the cautious interpretation of the findings was necessary. Colombini et al. (2021) carried out a systematic mini-review on hand movement sensing for neurocognitive disorders. The results showed that the use of VR in dementia seemed to be effective in the assessment and screening of functional abilities in cognitive impairment. However, empirical evidence was considered to still be limited, and deeper investigations were required. Jonson et al. (2021) reviewed the evidence surrounding the use of VR in the screening and diagnosis of spatial memory impairment and analyzed the potential limitations and compared them with standard neuropsychological tests. The authors critically discussed the evidence regarding the potential use of VR in rehabilitation, which is still uninvestigated. Kim et al. (2022) quantified the effect of VR as a therapeutic intervention for cognitive functions in individuals with MCI. Six studies involving 279 participants were examined. A significant improvement in cognitive function was recorded. Nevertheless, no improvement in the subcategories such as global cognition, working memory, executive functions, and attention was observed. Feedback stimulation through the use of VR was considered promising in the improvement of cognitive functioning in persons with MCI. A personalized VR program was viewed as useful for the enhancement of cognitive function. Li et al. (2022) conducted a systematic review of computerized cognitive training (CCT) in individuals with MCI. Overall, 18 studies with 1,059 participants were reviewed. A significant but small improvement in global cognitive behavior was recorded. The small sample and the short treatment duration were considered limitations of the studies. More comprehensive trials were warranted. The results of the detailed reviews were mixed. The remaining challenges include but are not limited to suitability in daily settings, sustainability, and affordability for families, professionals, and caregivers.

Mild cognitive impairment evidence was 6.7% in people aged between 60 and 64 years, 8.4% in the age group 65–69 years, 10.1% in the age group between 70 and 74 years, 14.8% in those aged 75–79 years, and 25.2% in the 80–84 years old group. Cumulative dementia incidence was 14.9% in individuals with MCI older than 65 years of age followed by 2 years. No high-quality evidence exists to support pharmacological treatment for MCI. In patients with MCI, exercise training (6 months) is likely to improve cognitive measures and cognitive training may improve cognitive measures (Petersen et al., 2018).

In light of the above, in the current opinion study, we postulate on the combination of RL and VR as a crucial means for assessment and recovery functions in MNI at an early stage of dementia. Specifically, we argue on the opportunity to combine both strategies and differentiate between normal aging–cognitive decline and MNI to prevent dementia stages or diseases. Moreover, we critically discuss the possibility of cognitive rehabilitation through both strategies combined in a unique approach, and we emphasize the effects of a combined intervention on the patients' quality of life and caregivers' burden reduction (a novelty feature of the opinion study). In the following sections, we concisely present both strategies separately, and propose the combination and/or the integration of both as a unique rehabilitative approach that could help in pursuing objectives related to both assessment and recovery. Finally, we argue the implications for both research and practice, and present some insights for future perspectives in this specific framework.

## Virtual reality

Virtual reality creates a computerized-based immersive environment that allows real-time interaction with users and provides a high degree of ecological validity. VR applications can be customized to meet specific clinical conditions and/or research needs. VR setups can be used to pursue dual crucial goals, namely (a) the assessment and (b) the recovery of cognitive functions. Once combined with serious games, VR has the basic merit of collecting real-time data from immersive users while those individuals can simultaneously be assessed with neuroimaging techniques such as functional magnetic resonance, positron emission tomography, and/or electroencephalography (Cabinio et al., 2020; Iliadou et al., 2021; Isernia et al., 2021).

VR represents an emerging technology-mediated intervention that digitally provides a three-dimensional artificial environment, enabling individuals to profitably interact ensuring the participants with sensorial inputs and/or track modifications. There are two main categories of VR, including fully immersive (i.e., involving a high level of immersion) and non-immersive environments (i.e., low levels of user immersion). The immersion provides the user with a sense of presence in the virtual world with an immersive device (e.g., head-mounted display) and an interactive tool or equipment (e.g., a joystick or a glove). VR has been adopted in healthcare and education for both assessment and rehabilitative goals. It represents an innovative approach to minimizing the negative impact of MNI on patients, families, professionals, and caregivers (Moreno et al., 2019; Gates et al., 2020).

VR has successfully been implemented and used in older adult populations. The enhancement of daily activities has been empirically observed in persons at high risk of cognitive impairment or decline. In addition, it has been positively used to reduce anxiety in older adults and NCI. The diagnosis of cognitive decline, caregiver education for major NCI, and executive functions in patients with stroke or acquired brain injuries have been profitably evaluated. However, empirical evidence is still sparse (Moreno et al., 2019; Jin et al., 2020).

## Reinforcement learning as part of machine learning

Several cognitive training tools have been developed for rehabilitative purposes to date. Such tools can be effective in older adult populations. Typically, a cognitive training program is constituted by sessions and is conducted by a multidisciplinary team (i.e., psychologist, occupational therapist, speech/language therapist, and neurologist). Practical exercises in different cognitive domains (e.g., attention, memory, and language) are usually carried out. Next to rehabilitative objectives, cognitive training can be adopted to improve mental performance in healthy people. Otherwise, it can be used to enhance academic performance in students or cognitive reserve in older adult populations (Cespón et al., 2015; Rai et al., 2021; Woolford et al., 2021).

Progressively and/or continuously adapting to the difficulty of the exercises and tasks based on individuals' capacities and competencies may be viewed as critical for the success of a rehabilitative program. Accordingly, current computerized systems commonly include specific mechanisms with graded difficulties along with sessions, exercises, and tasks. Usually, such difficulties increase gradually according to predefined rules assessed by a neuropsychologist who evaluates the achieved tasks. For instance, such computerized systems commonly adjust and increase the difficulty whenever a determined threshold is reached. However, the order in which the parameters characterizing the task are varied is fixed. Therefore, the relevance for the single trainee is still ignored. Thus, the specificities of the trainees are neglected (Zini et al., 2022).

An artificial intelligence-based adaptive mechanism may be considered reinforcement learning (RL) as part of machine learning (Cutler et al., 2021; Lee et al., 2021; Su et al., 2022). Broadly speaking, the general idea is that in computerized systems, an RL agent is associated with a single trainee for each exercise or task to be performed, that is, the agent interacts with the trainee while the exercise or task is executed and, based on RL peculiarities and algorithms, the agent is capable of learning from that specific experience that customized computerized cognitive systems. A tailored policy for each trainee will be pursued according to their ongoing performance. Specifically, the individual's performance while completing the task is used as a highly motivated reward to gradually solicit and stimulate a policy that it is useful to vary. Over time, the values of the parameters define the difficulty so that the trainee's performance is optimized in the long term. Consequently, the task can be processed individually and the policy adopted to modify its parameters to optimize the cognitive training can be learned autonomously for each trainee (Zini et al., 2022).

## Combined RL and VR

In light of the abovementioned ideas, one may consider the combined integration between RL and VR. It may be viewed as advantageous because the AI agent may calibrate and customize the difficulty of the exercises and/or tasks on the user, while the VR setup may provide an immersive environment similar to real life. The integration of different technologies has been recently postulated and suggested by Stasolla et al. (2022, 2023). In addition to the beneficial impact for the user, families and caregivers may additionally experience positive outcomes (Varey et al., 2021; Chou et al., 2022). The novelty feature is represented by the combined integration of both technologies whose application and implementation may be adopted for both assessment and rehabilitative purposes in the early cognitive decline among healthy populations on the one side and may be helpful in the early stage of the MNI as predictive of future dementia (Diaz-Orueta et al., 2020; Fields et al., 2021; Lisko et al., 2021; Otake-Matsuura et al., 2022).

Specifically, an ideal starting point for an artificial agent working according to RL principles as detailed above usually begins from a fully randomized policy criterion. Thus, an initial identical probability is assigned to all considered actions that the agent can carry out. That is, all the similar probabilities executed are preliminarily recognized in all the exercises/tasks. Two different outcomes are acknowledged: (a) a successful consequence is empirically observed because an optimal policy has been planned with a long interaction between the user and the system, or (b) a failure is recorded because an initial poor effect of the learning process was programmed, and frustration or demotivation by the user is recorded (Zini et al., 2022). To overcome this issue, one may envisage an association between category policies and exercises or tasks. The category policy should include a probability of actions that is not the ideal one but very close to it if compared to the fully randomized policy. Beginning from the task category policy, a tailored user-agent interaction would be developed, and the fineness of the ideal individual-customized policies can be programmed (Nowakowski et al., 2021).

VR environments can provide great value to immersive situations similar to those of real life. Older adults involved in VR setups may be exposed to experimental conditions with a high ecological validity with a dual objective: (a) assessment and (b) recovery of cognitive functioning. Both healthy individuals and persons with MNI may be evaluated and diagnosed with a prognostic goal. A customized rehabilitative intervention to prevent the negative consequences of cognitive decline may be designed (Tuena et al., 2021, 2022; Bacanoiu and Danoiu, 2022). Combined with an AI RL agent, VR may be constantly adapted and adjusted to the user, even during a working session, with positive outcomes for users, families, and caregivers (Jiang et al., 2021). To the best of our knowledge, such integration is still uninvestigated although warranted.

Similar to computerized-based programs, a combined setup including VR and RL may provide MNI participants with daily preferred activities or items in which the participant would ask for a desired or needed activity or option through the mediation of a caregiver. The AI agent will respond to the participant's performance and adjust the task difficulty accordingly. The caregiver may be involved in assessing the hierarchical system (i.e., multiple options proposed to be selected with a unique behavioral response produced by the participant), and the difficulty may be adjusted continuously through the AI and RL-based system. In addition, one may envisage remote supervision such as telerehabilitation (Bernini et al., 2021; Nebot et al., 2022).

## Discussion

Mild neurocognitive impairment may be considered a first sign of cognitive decline. An early evaluation may be viewed as crucial for any assessment or recovery of cognitive functioning. Furthermore, a healthy population may benefit greatly from early and comprehensive healthcare. Recently, new technologies have increasingly been developed. Next to VR, AI has successfully been implemented to improve the quality of life of young and older healthy adults. Commonly, one technology-based strategy has been adopted (Jin et al., 2020; Rocha et al., 2022).

Individuals with MNI may be involved in immersive virtual conditions similar to real life. For instance, Liu et al. (2023) divided 30 participants with mild post-stroke cognitive impairment into two groups, a group that used immersive VR-based puzzle game therapy and a control group exposed to traditional cognitive training. Montreal Cognitive Assessment (MoCA) was administrated before and after the treatment. Funny and serious games, including life skills, were implemented. Mondellini et al. (2022) exposed 15 older adults diagnosed with MCI to a virtual supermarket. The participants were requested to do the shopping, picking the item presented in a list and putting them in a cart. Appel et al. (2020) enrolled 66 participants with different cognitive functioning in an immersive VR setup that included 3–20 min of 360°-video footage of a nature scene displayed on a Gear VR HMD. Participants tried the VR setup at the medical center with their caregivers. Liao et al. (2019) recruited 34 community-dwelling older adults with MCI to undertake randomized VR-based physical and cognitive training or combined traditional physical and cognitive training. Thirty-six sessions were collected over 12 weeks. Outcome measures included executive functions, trail-making tests, gait performance, and dual-task costs. The results demonstrated an improvement in executive functions and single-task in both groups. However, the VR condition showed improvements in the dual-task condition and gait performance. In addition to aging–cognitive decline association (Deary et al., 2009), aging and the concomitant development of non-neuro-cognitive disorders due to lifestyles lead to cognitive decline (Zarnani et al., 2019; Xu et al., 2023).

Here, we examined the use of an RL AI agent combined with VR. We have argued and detailed a hypothesis that could be used as a successful strategy for assessment and rehabilitative objectives. Both healthy individuals and people with MNI may be targeted. One may emphasize the combined integration of both technologies as a relevant diagnostic tool (Isernia et al., 2021). Otherwise, a rehabilitative technology-based program may be implemented (Lancioni et al., 2020). One may additionally argue that the participant is constructively engaged and positively occupied. Accordingly, isolation, passivity, behaviors that are challenging (e.g., wandering), and/or withdrawal could be profitably prevented (Lancioni et al., 2009, 2011). However, the user's satisfaction and experience should be carefully assessed (Bernini et al., 2023).

RL combined with VR can provide a continuous, highly customer-tailored adaptation to each user. Their combined integration may be viewed as a further powerful support for cognitive reserve in both healthy and MNI individuals (Caffò et al., 2016). Nevertheless, its sustainability should be rigorously evaluated. Thus, financial and human resources should be adequately supported. The user's capacity to be involved in the proposed technology-based combination should be constantly monitored. The suitability in daily contexts and/or environments should be considered. The outcomes on the participant's quality of life and both caregivers' and families' burdens should be daily addressed (Gates et al., 2020). The endorsement of professionals (i.e., clinicians and researchers) should be recorded through social validation procedures (Stasolla et al., 2019, 2021). The proposed assessment tool might be used in neuro-developmental disorders as well as in neurocognitive decline and among healthy individuals as a preventive evaluation (Banerjee and Chan, 2008; Bowman et al., 2019).

## Limitations and future research perspectives

Despite encouraging and promising perspectives, caution is mandatory, and the different limitations of this approach should be acknowledged. First, the proposed combined integration of RL and VR lacks empirical data. Systematic studies on both targeted populations (i.e., aged healthy individuals and MNI people) should be conducted. Group comparisons and longitudinal single-subject-based studies should be carried out. Second, both assessment and recovery purposes of cognitive functioning should be carefully addressed. Third, the ability and knowledge base of older adults, who may not necessarily be familiar with the technology should be addressed. Fourth, the suitability of VR being integrated into daily contexts (i.e., home, work, and/or medical/rehabilitative centers) should be a research priority. Fifth, affordability in daily contexts and settings should be carefully assessed. Sixth, the proposed tool should be carefully assessed through a large sample of participants, including healthy individuals and persons with cognitive decline for differentiating both targets.

In light of the above, future research perspectives should deal with the following topics: (a) they should carry out systematic empirical studies using combined RL and VR in both healthy and MNI populations, (b) studies should evaluate the suitability and sustainability of human, environmental, and financial resources, (c) research needs to evaluate the affordability of implementing these technologies through social validation procedures that involve psychologists, neurologists, clinicians, researchers, and caregivers as external and expert raters (Stasolla et al., 2014, 2018), and (d) progressive systematic reviews and meta-analysis should be conducted within this specific framework.

## Conclusion

This study has proposed the prospective combination of RL and VR in pursuing the dual goals of assessment and recovery in healthy and MNI individuals. The targeting of cognitive functioning is potentially interesting and highly relevant for either aging populations or MNI. Early assessment of patients using the combined technology could be crucial in preventing future dementia outcomes. Otherwise, the integrated technology can be used as a useful rehabilitative strategy, providing a continuous and rigorous customized adaptation of the task or activity difficulty. A systematic matching between different technologies (e.g., assistive technology, telerehabilitation, and VR) has also been recently recommended by Stasolla et al. (2023).

A careful assessment of the customized and tailored solution for each use is warranted. Remote supervised control might be used in applying these technologies. Specific adaptations, suitability evaluations, and satisfying experience and usability should be systematically addressed (Bernini et al., 2023). Future research and practice should progressively include combined and integrated technologies to evaluate and recover cognitive functioning in older populations and more generally, in neurological populations.

## Author contributions

FS conceived and drafted the paper. MDG edited and critically revised it. FS and MDG made an intellectual contribution to the manuscript and approved the final submitted version of the article.
